# A Self‐Healing Flexible Quasi‐Solid Zinc‐Ion Battery Using All‐In‐One Electrodes

**DOI:** 10.1002/advs.202004689

**Published:** 2021-02-14

**Authors:** Jinyun Liu, Jiawei Long, Zihan Shen, Xing Jin, Tianli Han, Ting Si, Huigang Zhang

**Affiliations:** ^1^ Key Laboratory of Functional Molecular Solids (Ministry of Education) Anhui Provincial Engineering Laboratory for New‐Energy Vehicle Battery Energy‐Storage Materials College of Chemistry and Materials Science Anhui Normal University Wuhu Anhui 241002 P. R. China; ^2^ National Laboratory of Solid State Microstructures College of Engineering and Applied Sciences Nanjing University Nanjing Jiangsu 210093 P. R. China; ^3^ Department of Modern Mechanics University of Science and Technology of China Hefei Anhui 230026 P. R. China

**Keywords:** all‐in‐one, flexible, hydrogel electrolyte, self‐healing, Zn‐ion battery

## Abstract

Self‐healing and flexibility are significant for many emerging applications of secondary batteries, which have attracted broad attention. Herein, a self‐healing flexible quasi‐solid Zn‐ion battery composing of flexible all‐in‐one cathode (VS_2_ nanosheets growing on carbon cloth) and anode (electrochemically deposited Zn nanowires), and a self‐healing hydrogel electrolyte, is presented. The free‐standing all‐in‐one electrodes enable a high capacity and robust structure during flexible transformation of the battery, and the hydrogel electrolyte possesses a good self‐healing performance. The presented battery remains as a high retention potential even after healing from being cut into six pieces. When bending at 60°, 90°, and 180°, the battery capacities remain 124, 125, and 114 mAh g^−1^, respectively, cycling at a current density of 50 mA g^−1^. Moreover, after cutting and healing twice, the battery still delivers a stable capacity, indicating a potential use of self‐healing and wearable electronics.

## Graphical abstract

A self‐healing flexible quasi‐solid Zn‐ion battery composes of all‐in‐one cathode and anode, and a self‐healing hydrogel electrolyte. The free‐standing all‐in‐one electrodes enable a high capacity and flexible transformation of the battery, while the hydrogel electrolyte possesses a self‐healing performance. Under bending at different angles, and cutting/healing many times, the batteries remain very stable, indicating a promising potential for broad applications.

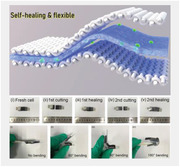

## Introduction

Self‐healing technology is developed rapidly in recent years, and has been applied in several fields including biological medicines, sensors, surface sciences, and intelligent functional materials.^[^ ^1^ ^]^ It realizes an automatic self‐repair function without manual operation, thereby improving the stability and long cycle life, as well as greatly reducing the maintenance cost. Recently, self‐healing has also become a hotspot for emerging energy storage systems. Many self‐healing batteries have been reported, such as the self‐healing Li‐ion, Na‐ion, and Mg‐ion batteries.^[^ ^2^ ^]^ For example, Zhao et al. reported a Li‐ion battery by using aligned carbon nanotube sheets loaded with LiMn_2_O_4_ and LiTi_2_(PO_4_)_3_ nanoparticles on a self‐healing polymer as electrode, while lithium sulfate/carboxymethylcellulose (Li_2_SO_4_/CMC) gel as both electrolyte and separator.^[^ ^3^ ^]^ The self‐healing performance was ascribed to the reconnection of hydrogen bonds and the van der Waals force on the surface of the breaking place. The battery delivered a capacity that decreased from 28.2 to 17.2 mAh g^−1^ at the rate of 50 mA g^−1^ after fifth cutting/self‐healing cycle. Zhong et al. reported a quasi‐solid‐state Na‐ion battery by using polyacrylamide (PAM) hydrogel as the electrolyte, which provided N and H atoms to form hydrogen bonds.^[^ ^4^ ^]^ Besides, self‐healing Mg‐ion battery was also successfully constructed. Among many self‐healing batteries, there are very few studies on the aqueous Zn‐ion battery with a self‐healing function. Zn‐ion batteries possess some attractive features such as high capacity, high abundance of Zn and low cost, high conductivity, low redox potential (−0.762 V vs standard hydrogen electrode), good safety,^[^ ^5^ ^]^ which enable them to be applied broadly such as in portable and wearable electronics, and large‐scale energy storage.

Many previous self‐healing investigations focus on the synthesis of self‐healing electrolytes. For instance, Wang et al. prepared a PAM hydrogel and immersed it in a solution containing ZnSO_4_ and MnSO_4_ to get a hydrogel electrolyte, which was used in a self‐healing Zn‐ion battery.^[^ ^6^ ^]^ After cutting/healing, the tensile stress of the hydrogel could maintain 66% and the Young's modulus was slightly raised on the basis of the hydrogen bonds between carboxyl groups. The *δ*‑MnO_2_ cathode‐based battery exhibited a capacity from 122 to 108 mAh g^−1^ after 100 cycles at a rate of 10 C. Moreover, Huang et al. prepared a poly(vinyl alcohol)(PVA)/Zn(CF_3_SO_3_)_2_ composite as the self‐healing electrolyte.^[^ ^7^ ^]^ The large amount of hydroxide (—OH) in PVA chains was able to form hydrogen bonds. After cutting/self‐healing four times, the battery delivered a capacity of 81.4 mAh g^−1^. Currently, it remains a big challenge to achieve a high self‐healing performance with compatible electrolyte and electrode systems.

Moreover, flexible batteries are of great significance to expand the applications of energy storage systems, which can be used widely in flexible electronics including wearable devices and robots.^[^ ^8^ ^]^ Some flexible substrates were employed as the current collectors. For example, Mo et al. deposited Ge‐ and N‐doped graphene on porous Ni foam to form an anode for Li‐ion batteries.^[^ ^9^ ^]^ Some other electrodes based on the substrates of carbon cloth (CC)/carbon fibers/stainless‐steel mesh/polypropylene membrane also displayed a flexible performance and maintained a good electrochemical performance after bending, twisting, or folding.^[^ ^10^ ^]^ Recently, constructing an emerging self‐healing flexible battery becomes a great interest. Once the battery is broken, it is able to repair spontaneously and keep a good flexibility simultaneously. Recently, a Zn‐ion battery reported by Lu et al. was able to recover after deformation on the basis of the reversible ionic bonds and hydrogen bonds between two polymers of gelatin and sodium alginate.^[^ ^11^ ^]^ By bending the battery into diameters of 10 and 30 mm, their V_2_O_5_/carbon nanotube batteries remained 86% and 89% of the initial capacity, respectively. Li et al. reported a Zn–MnO_2_ battery in which the electrolyte was carboxyl‐modified PVA. The self‐healing mechanism of the hydrogel was ascribed to the COO—Fe bonds.^[^ ^12^ ^]^ Ma et al. reported an all‐solid‐state Zn‐ion battery in which poly(vinylidene fluoride–hexafluoropropylene) (PVDF–HFP) and poly(ethylene oxide) (PEO) co‐polymer provided hydrogen bonds to enhance the flexibility.^[^ ^13^ ^]^ As seen from these achievements, it is promising to construct a high‐performance self‐healing flexible Zn‐ion battery by using hydrogel electrolyte and all‐in‐one 3D electrodes, which have been rarely studied.

**Figure 1:**
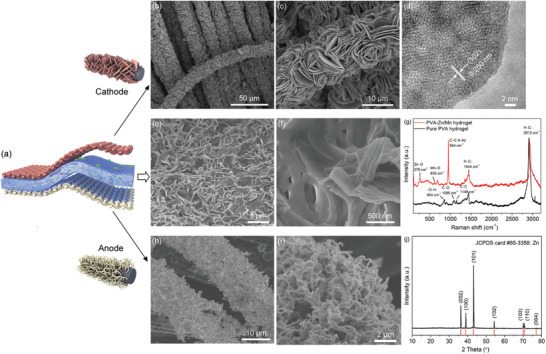
a) Illustration of the self‐healing flexible Zn‐ion battery. b) Low‐ and c) high‐magnification SEM images of the VS_2_ nanosheets/CC cathode. d) HRTEM image of a VS_2_ nanosheet. The lines at the bottom indicate the standard peak position. e) Low‐ and f) high‐magnification SEM images of the PVA–Zn/Mn hydrogel electrolyte. g) Raman shift of PVA–Zn/Mn hydrogel and the pure PVA hydrogel. h) Low‐ and i) high‐magnification SEM images of the Zn nanowires/CC anode. j) XRD pattern of the Zn/CC anode.

Here, in order to develop safe, portable, and wearable secondary battery systems, we present a novel self‐healing and flexible Zn‐ion battery composing of PVA/Zn(CH_3_COO)_2_/Mn(CH_3_COO)_2_ (named as PVA–Zn/Mn) hydrogel as electrolyte, VS_2_ nanosheets growing on CC as cathode, and Zn‐nanowire‐deposited CC as anode, as illustrated in **Figure** **1**a. The PVA–Zn/Mn hydrogel electrolyte possesses a large amount of —OH group on the PVA chains, which can easily form hydrogen bonds to achieve a self‐healing function. Dense VS_2_ nanosheets were in situ grown on CC through a hydrothermal method, forming a free‐standing cathode, while Zn anode was prepared by the electrochemical deposition of Zn nanowires on CC. The presented Zn‐ion battery displays both flexible and self‐healing properties. Furthermore, even though the battery was cut into six pieces, it can be healed quickly with a slight decay of the overall battery potential.

## Results and Discussion

VS_2_ nanosheets were hydrothermally grown on the conductive and flexible CC current collector, forming a free‐standing all‐in‐one VS_2_/CC cathode. The scanning electron microscopy (SEM) images of the VS_2_/CC cathode are shown in Figure 1b,c. The surface of the carbon fibers is covered by dense VS_2_ nanosheets, displaying a loading of about 15.1 mg cm^−2^. In Figure S1a (Supporting Information), the thickness of each nanosheet is about 180 nm. The transmission electron microscopy (TEM) images are displayed in Figure S1b,c (Supporting Information), and the high‐resolution TEM (HRTEM) image of a piece of VS_2_ (Figure 1d) shows a lattice spacing of 0.25 nm, corresponding to the (102) plane of the hexagonal phase of VS_2_. The selected‐area electron diffraction (SAED) pattern (Figure S1d, Supporting Information) displays a series of concentric rings which are indexed to the (001), (101), (102), and (110) planes of VS_2_, indicating a polycrystalline structure. The diffraction peaks in the X‐ray diffraction (XRD) pattern (Figure S1e, Supporting Information) are indexed to the hexagonal VS_2_ by referencing to the Joint Committee on Powder Diffraction Standards (JCPDS) card #36–1139. Moreover, the elemental mapping images (Figure S2a–d, Supporting Information) and the corresponding energy dispersive spectroscopy (EDS) spectrum (Figure S2e, Supporting Information) show that V and S distribute throughout the carbon fiber, while the line‐scan curves (Figure S2f, Supporting Information) confirm their uniform and dense distribution, which indicates a high VS_2_ loading within the all‐in‐one cathode.

The X‐ray photoelectron spectroscopy (XPS) survey spectrum (Figure S3a, Supporting Information) shows the composition of the VS_2_/CC cathode composing of elements of V, S, and C. The high‐resolution XPS spectrum of V 2p (Figure S3b, Supporting Information) displays the peaks at 524.6 and 517.2 eV, which are assigned to the V 2p_1/2_ and V 2p_3/2_ spin orbitals, respectively, confirming that the element V has a single valence of +4.^[^ ^14^ ^]^ In the S 2p spectrum (Figure S3c, Supporting Information), two peaks are located at 170.2 and 168.9 eV, corresponding to the sulfate generated from surface oxidation.^[^ ^15^ ^]^ The peaks at 164.4 and 163.5 eV are assigned to S 2p_3/2_, while a single peak at 161.2 eV is ascribed to S 2p_1/2_, which are corresponding to the S^2−^ phase.^[^ ^16^ ^]^ In addition, there is a peak at 289.1 eV in the XPS spectrum of C 1s (Figure S3d, Supporting Information), which is attributed to the C=O, while two peaks centered at 286.5 and 284.2 eV are assigned to C—C sp^2^ and C—C sp^3^/C—S, respectively.^[^ ^17^ ^]^

In order to take the SEM images of the hydrogel electrolyte, the PVA–Zn/Mn and pure PVA hydrogels were immersed in liquid nitrogen, then placed in a vacuum freeze‐drier for 2 days to completely remove water. In Figure 1e,f, after adding the Zn(Ac)_2_ and Mn(Ac)_2_ into the PVA hydrogel, the SEM images show a porous structure while the pure PVA hydrogel exhibits a dense profile in Figure S4 (Supporting Information), which is beneficial for the diffusion of Zn ions. In the Raman spectra (Figure 1g), the bands at 854, 1095, and 1149 cm^−1^ are assigned to the O—H, C—O, and C—C vibrations, respectively, while 1444 and 2913 cm^−1^ correspond to the C—H vibration, verifying the chain of PVA.^[^ ^18^ ^]^ The bands at 279, 635, and 954 cm^−1^ are indexed to the Zn—O, Mn—O, and C—C vibrations, respectively, confirming the existence of Zn and Mn ions.^[^ ^19^ ^]^ In the Fourier transform infrared spectroscopy (FTIR) spectra (Figure S5, Supporting Information), the board peak at 3442 cm^−1^ is ascribed to the adsorption of water on samples during measurement.^[^ ^20^ ^]^ The peaks at 2941, 1097, and 846 cm^−1^ of the pure PVA hydrogel are attributed to the —CH_2_ stretching, C—O—H bending, and C—C vibrations on the PVA chain, respectively, while 1445 and 1330 cm^−1^ are assigned to the C—O stretching vibration.^[^ ^21^ ^]^ After the metal salts were added, the new peaks at 1583 and 1025 cm^−1^ could be assigned to the —COOH asymmetric stretching and C—CH_3_ stretching vibrations, respectively.^[^ ^22^ ^]^ In addition, the SEM images of the anode by electrochemically depositing Zn on CC are shown in Figure 1h,i. Dense Zn nanowires were coated on the surface of the carbon fibers, resulting in an all‐in‐one Zn nanowires/CC anode. In Figure 1j, the XRD pattern confirms the Zn phase coated on the CC with a high purity.

**Figure 2:**
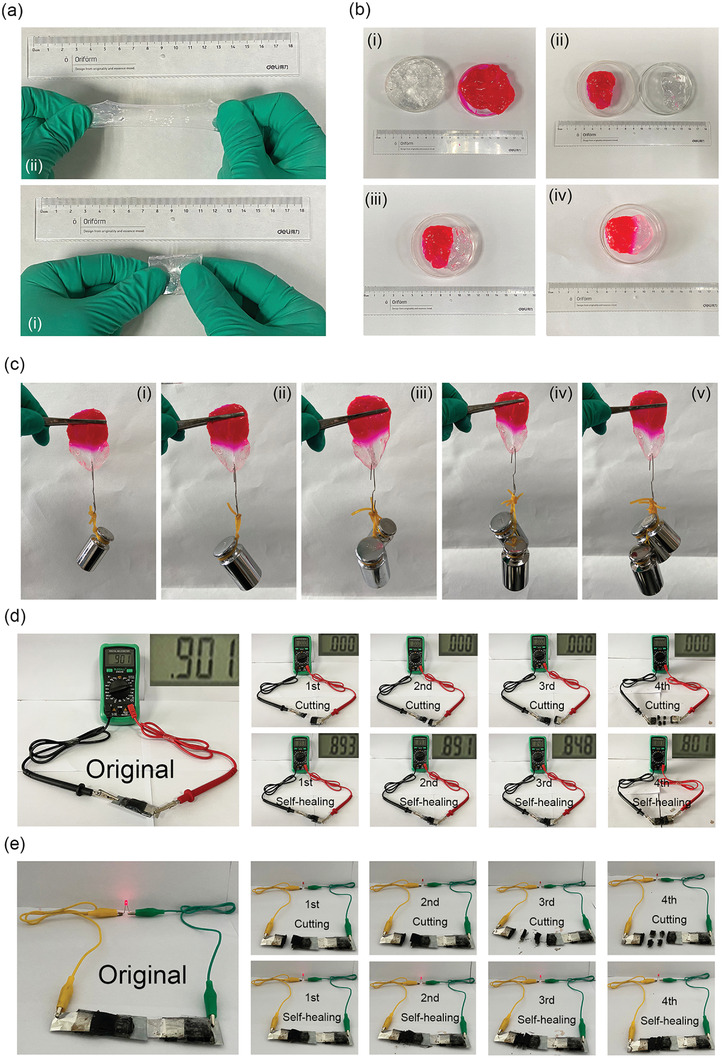
a) Photos of the PVA–Zn/Mn hydrogel electrolyte i) before and ii) after being stretched. b) Self‐healing demonstration: i) two pieces of hydrogels with different colors; ii) two half pieces; iii,iv) the two pieces contact for iii) 0 min and iv) 30 min. c) Weight‐bearing tests of the healed hydrogel with a series of loading weights of i) 100, ii) 200, iii) 300, iv) 400, and v) 500 g. d,e) Photographs of the self‐healing flexible Zn‐ion battery after first, second, third, and fourth times of cutting and self‐healing: d) battery potentials and e) LEDs lightened by the battery after each time of the self‐healing process.

The self‐healing performance of the hydrogel electrolyte is shown in **Figure** **2**. In Figure 2a, a piece of the PVA–Zn/Mn hydrogel with a length of 3 cm was elongated to about 12 cm without breaking, indicating a good flexibility. A video about the stretching process is demonstrated in Movie S1 (Supporting Information). Furthermore, two pieces of hydrogels with the same diameter of 5 cm were cut from the middle position, then each half was contacted and left for 30 min, as shown in Figure 2b(i)–(iv). The observations toward the cut sections indicate a similar morphology as the one before cutting (Figure 1e,f). It is found that two pieces adhere tightly. The tensile tests were conducted under a series of loading weights from 100 to 500 g, as displayed in Figure 2c. For example, at a loading of 500 g, the healed hydrogel keeps well. The self‐healing mechanism is ascribed to the formation of abundant hydrogen bonds between the hydroxyls in the PVA chains. Since that, the self‐healing performance would be affected mainly by the density of functional groups and the thickness of the film. In order to improve the self‐healing performance further, improving the functional groups within the hydrogel electrolyte and optimizing the thickness of hydrogel film would be valuable.

To evaluate the self‐healing properties of the Zn‐ion battery composing of the hydrogel electrolyte, VS_2_/CC cathode, and Zn/CC anode, a fully charged battery with the potential of 0.901 V was cut into different pieces. In the first, second, and third demonstrations, the battery was vertically cut, while in the fourth time, the battery was horizontally cut. In Figure 2d, it is found that potentials of the battery remain 0.893, 0.891, 0.848, and 0.801 V after each cut/self‐healing process. The potential retention is as high as 88.9% after each time of self‐healing. In addition, two batteries were connected in series to lighten a light‐emitting diode (LED), as displayed in Figure 2e. The LED was lightened even after four times of the cut/healing process, indicating a promising potential for practical self‐healing applications.

**Figure 3:**
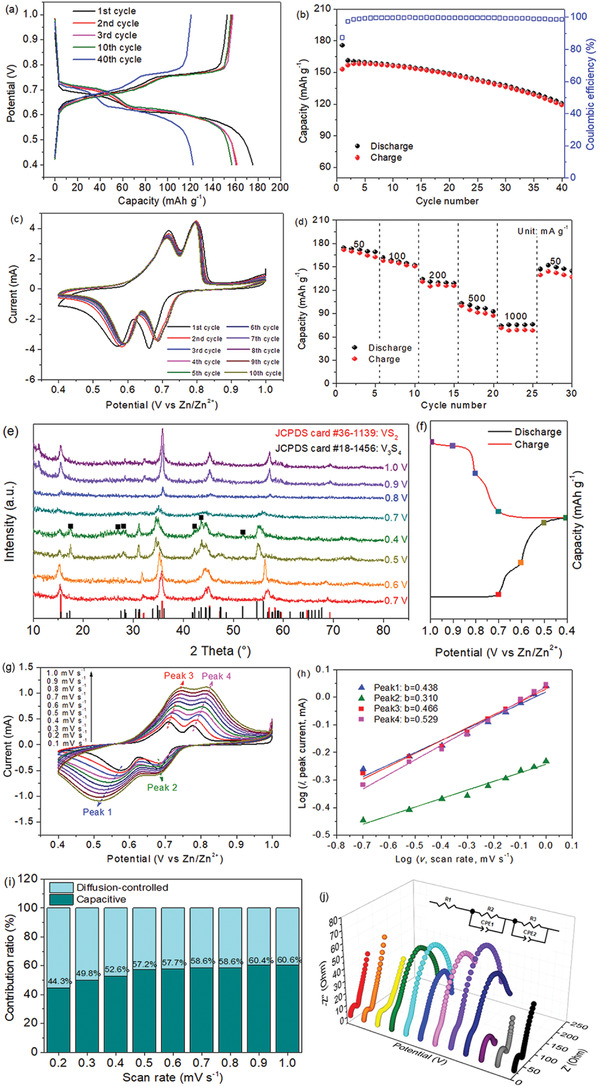
Electrochemical performance of the Zn‐ion batteries. a) Galvanostatic charge–discharge curves and b) cycling performance at 200 mA g^−1^. c) CV curves at 0.1 mV s^−1^. d) Rate performance. e) Ex situ XRD patterns of the VS_2_/CC cathode when charging–discharging at various potentials. f) Corresponding charge–discharge profile illustrates each potential position. g) CV curves at a series of rates from 0.1 to 1.0 mV s^−1^. h) The fitted lines of log(*i*) versus log(*v*) plots for different peaks. i) Ratios of the capacitive to diffusion‐controlled contributions. j) Nyquist plots of the battery within one charge–discharge cycle. The inset shows the equivalent circuit used for fitting.

**Figure** **3**a,b presents the cycling performance of the self‐healing Zn‐ion battery at a current density of 200 mA g^−1^. The plateaus at 0.7 and 0.6 V in discharge and the ones at 0.7 and 0.75 V during charge well match the redox peaks in cyclic voltammetry (CV) curves (Figure 3c), respectively. The discharge capacities at the 1st, 2nd, 3rd, 10th, and 40th cycles are 175, 161, 161, 157, and 123 mAh g^−1^, respectively. The initial Coulombic efficiency is 68%, which is attributed to the formation of solid–electrolyte interphase (SEI) film, while it becomes as high as 99.7% after 40 cycles. The capacity fade is ascribed to the passivation of Zn anode^[^ ^23^ ^]^ and the formation of Zn(OH)_4_^2−^ and ZnO, which further prevent the reactions with Zn.^[^ ^24^ ^]^ The SEM images of the postcycled cathode verify a robust structure, as shown in Figure S6 (Supporting Information). In addition, Figure S7a,b (Supporting Information) shows the cycling performances at 50 mA g^−1^ of the batteries based on the all‐in‐one VS_2_/CC cathode and a slurry‐coated VS_2_ cathode using carbon paper as substrate. For comparison, the dispersive VS_2_ nanosheets were also prepared through the similar hydrothermal route without using CC as substrate. The cathode was constructed with VS_2_ nanosheets, carbon black, and sodium carboxymethylcellulose binder. The battery based on the VS_2_ nanosheet cathode shows a much low discharge capacity of 44 mAh g^−1^, which is 96 mAh g^−1^ for the VS_2_/CC cathode after 60 cycles, indicating the significance of growing VS_2_ onto 3D CC to form a free‐standing all‐in‐one electrode. Furthermore, the VS_2_/CC cathode with a relatively high VS_2_ loading of 25.3 mg cm^−2^ was also prepared, which showed a capacity of 102 mAh g^−1^ at 50 mA g^−1^, as displayed in Figure S8 (Supporting Information). Figure 3c shows the CV curves at the first ten cycles in the potential range of 1.0–0.4 V versus Zn/Zn^2+^ at a scanning rate of 0.1 mV s^−1^. Two reduction peaks at 0.58 and 0.68 V correspond to the Zn^2+^ insertion, while the anodic peaks at 0.72 and 0.8 V are assigned to the Zn^2+^ extraction. Figure 3d displays the rate performance at different current densities. The capacities are 162, 152, 125, 87, and 75 mAh g^−1^ at 50, 100, 200, 500, and 1000 mA g^−1^, respectively. The capacity recovers to 146 mAh g^−1^ when the rate is back to 50 mA g^−1^. The capacity decay between the first and the second time cycling at 50 mA g^−1^ is 9.8%.

Figure 3e displays the ex‐situ XRD patterns of the all‐in‐one VS_2_/CC cathode at the selected potentials indicated in Figure 3f. During the discharge from 0.7 to 0.6, 0.5, and to 0.4 V, the peaks located at 15.4°, 32.1°, 35.7°, 45.2°, and 57.1° correspond to the (001), (100), (101), (102), and (110) planes of VS_2_. It is noted that there is a slightly negative shift, which is ascribed to the gradual Zn^2+^ insertion in the interlayer spacing of VS_2_, forming a Zn*_x_*VS_2_.^[^ ^25^ ^]^ At 0.4 V, some new peaks at 17.3°, 27.5°, 28.3°, 42.3°, 44.4°, and 51.9° appear as marked by black squares, which are attributed to the formation of V_3_S_4_ with continuous insertion of Zn^2+^ ions. The V(IV) is reduced depending on the insertion of Zn^2+^ ions, which leads to the phase transformation to Zn*_x_*V_3_S_4_. In the subsequent charging process, these peaks become weak and finally disappear at 1.0 V. Most of the V_3_S_4_ returns to VS_2_, resulting in that the XRD pattern at 1.0 V can be indexed to the VS_2_. The reaction in charge and discharge processes is presented as 4VS_2_ + (*x* + *y*) Zn^2+^ + 2 (*x* + *y*) e^−^↔ Zn*_x_*V_3_S_4_ + 2S, where the value of *x* + *y* is calculated as 0.4 on the basis of the discharge capacity, as shown in Figure S9 (Supporting Information) with more details.

The CV curves at various of scan rates from 0.1 to 1.0 mV s^−1^ are displayed in Figure 3g. The shape of the CV profiles keeps close along with a slightly shifting of each peak, which is ascribed to the polarization under the increasing rates. The capacitive and diffusion‐controlled behaviors are investigated by equation *i* = a*v^b^*, where *i* stands for the current density, *v* presents the scan rate, and *a* and *b* are the adjustable parameters. It is transformed to equation log(*i*) = *b*log(*v*) + log(*a*), where the value of *b* is determined by the slope of log(*i*) and log(*v*). When *b* = 0.5, it is related to a diffusion‐controlled process, whereas *b* = 1 reflects a capacitive behavior.^[^ ^26^ ^]^ In Figure 3h, the *b* values of peaks 1, 2, 3, and 4 are 0.438, 0.310, 0.466, and 0.529, respectively, indicating both capacitive and diffusion‐controlled behaviors of the battery. Moreover, the contribution ratios at various sweep rates can be calculated by *k*_1_ *v* and *k*_2_ *v* ^1/2^ according to the equation *i*(V) = *k*_1_ *v* + *k*_2_ *v* ^1/2^ and *i*(V)/*v* ^1/2^ = *k*_1_ *v* ^1/2^ + *k*_2_ *v*,^[^ ^27^ ^]^ where *k*_1_ and *k*_2_ are constants. The *k*_1_ and *k*_2_ can be calculated by plotting the fitting lines of *i*(V)/*v* ^1/2^ and *k*_2_ *v*. Since that, the capacitive contribution (*k*_1_ *v*) and diffusion‐controlled contribution (*k*_2_ *v* ^1/2^) at various scan rates are obtained, as shown in Figure 3i. The capacitive contribution increases with the increase of scanning rates. The electrochemical impedance spectra (EIS) at a series of potentials within one cycle are shown in Figure 3j. The fresh cell was discharged from 0.9 to 0.4 V, and then charged back to 1.0 V. It exhibits an increasing trend of charge‐transfer resistance when discharging to 0.4 V. The fitted resistances are shown in Table S1 (Supporting Information). The oblique line disappears at the discharge potential of 0.6 V. It is ascribed to the gradually formation of sulfur, which reduces the conductivity. The subsequent charging processes show a decreasing trend of charge‐transfer resistance, which is attributed to the decrease of sulfur component.

**Figure 4:**
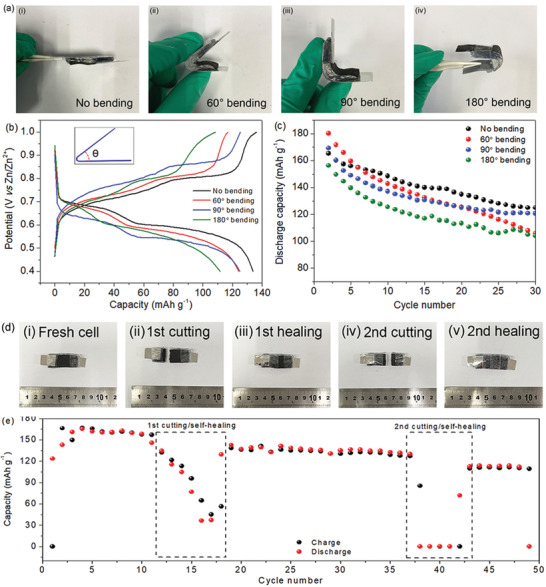
a) Photos of the self‐healing flexible Zn‐ion battery after bending to different angles: i) no bending, ii) 60°, iii) 90°, and iv) 180°. b) Galvanostatic charge–discharge profiles of the battery under different bending states after cycling 30 times at 50 mA g^−1^. The inset shows the illustration of the bending angle. c‐i–v) Corresponding discharge capacities of the batteries. d) Photo of the battery after cutting and healing and e) the corresponding cycling performance at 50 mA g^−1^.

The self‐healing flexible battery before and after bending to 60°, 90°, and 180° was also cycled at a current density of 50 mA g^−1^, as illustrated in **Figure** **4**a(i)–(iv). The corresponding performance is shown in Figure 4b,c. The battery without bending and bending at 60°, 90°, and 180° deliver capacities of 165, 180, 169, and 156 mAh g^−1^, which remain 123, 110, 121, and 106 mAh g^−1^ after 30 cycles, respectively. The decay with the increase of bending angle is attributed to the compressive and shear stresses on the battery system, which would impact the interfacial contact and properties.^[^ ^28^ ^]^ It indicates the good flexibility of the fabricated battery in addition to the self‐healing property, indicating its promising application in wearable electronics, self‐healing intelligent devices, etc. In addition, the cutting and healing performances were also conducted during the cycling process. In Figure 4d, the battery before cutting was initially cycled ten times. During the cutting, the electrodes and hydrogel were separated into two pieces. Then the two pieces were put together for contact, and the overall battery was packaged again by an adhesive polypropylene film. During the cutting process, the PVA chains would break and some free‐moving PVA chain segments would expose on the fresh fracture surface. After the recontact (healing), the PVA chains would attract each other by hydrogen bonds, re‐forming a hydrogen bonding,^[^ ^5^ ^]^ which makes the two pieces of hydrogel heal as a whole film again, as illustrated in Figure S10 (Supporting Information). In addition, the separated electrodes recontact by the adhesive effect of electrolyte. In our study, the self‐healed battery was placed for 6 h before subsequent cycling tests. The corresponding cycling performance is displayed in Figure 4e. The fresh cell exhibits a capacity of 158 mAh g^−1^ in the 10th cycle, and soon decays after the first cut‐healing treatment. However, the capacity gradually recovers back to 142 mAh g^−1^ in the 19th cycle, which is ascribed to the accomplished self‐healing. The second time of cutting–healing was conducted at the 37th cycle. It shows almost no capacity at the next five cycles. Nevertheless, then the capacity recovers back to 112 mAh g^−1^, which is close to the first‐round cutting–healing process. At last, the battery can be cycled normally after the cutting–healing treatments, indicating a potential for applications in many fields.

In summary, we present a self‐healing flexible aqueous Zn‐ion battery, which consists of a PVA–Zn/Mn electrolyte, all‐in‐one 3D VS_2_/CC cathode, and Zn/CC anode. The constructed Zn‐ion battery possesses both flexible and self‐healing performances. After cycling at a rate of 200 mA g^−1^ 40 times, the battery exhibits a stable capacity of 123 mAh g^−1^. After bending for 60°, 90°, and 180°, the battery capacities remain 110, 121, and 106 mAh g^−1^ after 30 cycles. Moreover, even though the battery was cut into six pieces, it was able to self‐heal quickly with only a little decay of the overall potential of the battery, indicating a good self‐healing stability. During charge–discharge, the battery was cut into two pieces; after self‐healing, it delivered a recoverable capacity compared to the one before cutting. It is expected that the presented self‐healing flexible Zn‐ion battery could find important applications for flexible electronics and self‐healing supplies. Of course, the cycling performance at a high current density still needs further investigation. It could be enhanced by improving the Zn‐ion concentration and ionic conductivity of the hydrogel electrolyte, which would have some interesting directions in future. Moreover, the all‐in‐one electrodes including both anode and cathode would be applicable for constructing a broad set of other emerging energy‐storage systems.

## Experimental Section

##### Preparation of the Electrodes

At first, a free‐standing VS_2_ nanosheets/CC cathode was prepared through a solvothermal approach. All chemicals were analytical reagent grade purchased from Aladdin and used without purification. Typically, 3.6 mmol of NH_4_VO_3_ and 27 mmol of thioacetamide were dissolved in 30 mL of deionized water, followed by the addition of 3.6 mL of NH_3_·H_2_O (37 wt%). After continuously stirring for 1 h, a piece of CC was immersed in the solution under ultrasonication, then the mixture was poured into a polytetrafluoroethylene (PTFE)‐lined stainless‐steel autoclave and sealed. The autoclave was placed in an oven at 180 °C for 8 h. After that, the CC growing with VS_2_ nanosheets was collected and washed by water and ethanol then dried at 60 °C under vacuum. The VS_2_ loading of the cathode was calculated by accurately weighing the mass of CC before and after VS_2_ growth.

Zn nanowires were prepared on the surface of CC via electrodeposition using a three‐electrode system, where the carbon cloth, a platinum wire, and a saturated calomel electrode were served as the working electrode, counter electrode, and reference electrode, respectively. Electroplate solution was prepared by dissolving 6.25 g of ZnSO_4_·7H_2_O, 6.25 g of Na_2_SO_4_, and 1 g of H_3_BO_3_ in 50 mL of deionized water under magnetic stirring. Subsequently, zinc was electroplated on substrate for 30 min under a constant current density of −40 mA cm^−2^.

##### Preparation of Self‐Healing Hydrogel Electrolyte

The self‐healing hydrogel was prepared by dissolving 13 g of PVA in a 50 mL solution containing 12 g of Zn(CH_3_COO)_2_·2H_2_O and 1.225 g of Mn(CH_3_COO)_2_·4H_2_O under slowly magnetic stirring at 85 °C. After 1 h, the sticky solution was ultrasonicated to eliminate the small bubbles. Then, the solution was poured into a Petri dish and placed at room temperature overnight, forming a PVA–Zn/Mn hydrogel electrolyte. For comparison, pure PVA hydrogel was prepared by dissolving 13 g of PVA in 50 mL of deionized water then slowly stirred at 85 °C for 1 h.

##### Characterization

The morphology, structure, and composition were characterized by using SEM (Hitachi S‐8100, operated at 5 kV), TEM, Hitachi HT7700), HRTEM (Tecnai G220S‐TWIN/FEI), and XRD (Bruner D8 Advance) with the Cu K*α* radiation at a wavelength of 1.5418 Å. XPS (ESCALAB 250) was used to analyze the composition. The hydrogels were treated by liquid nitrogen before freeze–drying for 48 h to obtain the porous structure, then FTIR spectroscopy (IR‐21IR‐21) and Raman spectrometry (Renishaw, inVia) were used to study the groups of the PVA–Zn/Mn and pure PVA hydrogels.

##### Battery Construction and Electrochemical Tests

To study the flexibility and cutting/self‐healing performances, the quasi‐solid Zn‐ion batteries were assembled by using the as‐prepared PVA–Zn/Mn hydrogel electrolyte, VS_2_/CC cathode, and Zn/CC anode. The battery was packaged by adhesive polypropylene film. All procedures were carried out in an air surrounding. The bending angles were 60°, 90°, and 180°. The potentials of the batteries were measured by using a digital LCD display multimeter (Elecall, EM33D) before and after cutting/healing process. The photographs of a light emitting diode (LED) were taken after cutting and healing. In addition, for comparison, a conventional 2032‐typed coin cell was also assembled by using the prepared electrodes and a glass fiber separator. The electrolyte was prepared by dissolving 21.95 g of Zn(CH_3_COO)_2_·2H_2_O and 2.45 g of Mn(CH_3_COO)_2_·4H_2_O in 100 mL of water. The galvanostatic charge–discharge was conducted on a battery tester (Neware, CT‐4008). The capacities were measured at current densities of 50 and 200 mA g^−1^, respectively. The rate performance was tested at 50, 100, 200, 500, and 1000 mA g^−1^ then back to 50 mA g^−1^. Both the CV and EIS were measured on an electrochemical workstation (Chenhua, CHI‐660E).

##### Statistical Analysis

The illustration models were constructed on a Maya software (Autodesk, San Rafael, CA, USA). The images were processed on a Fireworks software (Adobe, San Jose, CA, USA). The XRD, XPS, HRTEM, Raman, FTIR, and EIS spectra were analyzed on Jade software (Materials Data, Livermore, CA, USA), Peak Fit software (Systat software, Inc, San Jose, CA, USA), Digital Micrography software (Gatan Inc, Pleasanton, CA, USA), Labspec software (Horiba, Kyoto, JP), OMNIC (Thermo Fisher Scientific Inc, Waltham, MA, USA), and ZView (Scribner Associates, Inc, Charlottesville, VA, USA), respectively. Results were analyzed on an OriginPro software (Origin Lab, Northampton, MA, USA).

## Conflict of Interest

The authors declare no conflict of interest.

1a) 
D. G.
Bekas
, 
K.
Tsirka
, 
D.
Baltzis
, 
A. S.
Paipetis
, Composites, Part B
2016, 87, 92;b) 
Y.
Han
, 
X.
Wu
, 
X.
Zhang
, 
C.
Lu
, Adv. Mater. Technol.
2019, 4, 1800424;c) 
D. L.
Taylor
, 
M.
in het Panhuis
, Adv. Mater.
2016, 28, 9060;.2748882210.1002/adma.201601613d) 
Z.
Wang
, 
L.
Scheres
, 
H.
Xia
, 
H.
Zuilhof
, Adv. Funct. Mater.
2020, 30, 1908098.2

A.
Du
, 
H.
Zhang
, 
Z.
Zhang
, 
J.
Zhao
, 
Z.
Cui
, 
Y.
Zhao
, 
S.
Dong
, 
L.
Wang
, 
X.
Zhou
, 
G.
Cui
, Adv. Mater.
2019, 31, 1805930.10.1002/adma.201805930306720393

Y.
Zhao
, 
Y.
Zhang
, 
H.
Sun
, 
X.
Dong
, 
J.
Cao
, 
L.
Wang
, 
Y.
Xu
, 
J.
Ren
, 
Y.
Hwang
, 
I. H.
Son
, 
X.
Huang
, 
Y.
Wang
, 
H.
Peng
, Angew. Chem., Int. Ed.
2016, 55, 14384.10.1002/anie.201607951277307534

L.
Zhong
, 
Y.
Lu
, 
H.
Li
, 
Z.
Tao
, 
J.
Chen
, ACS Sustainable Chem. Eng.
2018, 6, 7761.5a) 
D.
Selvakumaran
, 
A.
Pan
, 
S.
Liang
, 
G.
Cao
, J. Mater. Chem. A
2019, 7, 18209;b) 
H.
Jia
, 
Z.
Wang
, 
B.
Tawiah
, 
Y.
Wang
, 
C.‐Y.
Chan
, 
B.
Fei
, 
F.
Pan
, Nano Energy
2020, 70, 104523;c) 
G.
Fang
, 
J.
Zhou
, 
A.
Pan
, 
S.
Liang
, ACS Energy Lett.
2018, 3, 2480;d) 
S.
Liu
, 
L.
Kang
, 
J. M.
Kim
, 
Y. T.
Chun
, 
J.
Zhang
, 
S. C.
Jun
, Adv. Energy Mater.
2020, 10, 2000477.6

D.
Wang
, 
L.
Wang
, 
G.
Liang
, 
H.
Li
, 
Z.
Liu
, 
Z.
Tang
, 
J.
Liang
, 
C.
Zhi
, ACS Nano
2019, 13, 10643.3141938010.1021/acsnano.9b049167

S.
Huang
, 
F.
Wan
, 
S.
Bi
, 
J.
Zhu
, 
Z.
Niu
, 
J.
Chen
, Angew. Chem., Int. Ed.
2019, 58, 4313.10.1002/anie.201814653306979658a) 
L.
Li
, 
Z.
Lou
, 
D.
Chen
, 
K.
Jiang
, 
W.
Han
, 
G.
Shen
, Small
2018, 14, 1702829;10.1002/smll.20170282929164773b) 
T.
Yang
, 
D.
Xie
, 
Z.
Li
, 
H.
Zhu
, Mater. Sci. Eng., R
2017, 115, 1;c) 
J.
Kim
, 
R.
Kumar
, 
A. J.
Bandodkar
, 
J.
Wang
, Adv. Electron. Mater.
2017, 3, 1600260.9

R.
Mo
, 
D.
Rooney
, 
K.
Sun
, 
H. Y.
Yang
, Nat. Commun.
2017, 8, 13949.2805106510.1038/ncomms13949PMC521610110a) 
J.
Pan
, 
Y. Y.
Xu
, 
H.
Yang
, 
Z.
Dong
, 
H.
Liu
, 
B. Y.
Xia
, Adv. Sci.
2018, 5, 1700691;10.1002/advs.201700691PMC590837929721418b) 
Z.
Wang
, 
H.
Li
, 
Z.
Tang
, 
Z.
Liu
, 
Z.
Ruan
, 
L.
Ma
, 
Q.
Yang
, 
D.
Wang
, 
C.
Zhi
, Adv. Funct. Mater.
2018, 28, 1804560.11

Y.
Lu
, 
T.
Zhu
, 
N.
Xu
, 
K.
Huang
, ACS Appl. Energy Mater.
2019, 2, 6904.12

Q.
Li
, 
X.
Cui
, 
Q.
Pan
, ACS Appl. Mater. Interfaces
2019, 11, 38762.3158387910.1021/acsami.9b1355313

L.
Ma
, 
S.
Chen
, 
N.
Li
, 
Z.
Liu
, 
Z.
Tang
, 
J. A.
Zapien
, 
S.
Chen
, 
J.
Fan
, 
C.
Zhi
, Adv. Mater.
2020, 32, 1908121.10.1002/adma.2019081213209114914a) 
J.
Xu
, 
Y.
Zhu
, 
B.
Yu
, 
C.
Fang
, 
J.
Zhang
, Inorg. Chem. Front.
2019, 6, 3510;b) 
L.
Cai
, 
Q.
Zhang
, 
J. P.
Mwizerwa
, 
H.
Wan
, 
X.
Yang
, 
X.
Xu
, 
X.
Yao
, ACS Appl. Mater. Interfaces
2018, 10, 10053.2949850310.1021/acsami.7b1879815a) 
D.
Wu
, 
C.
Wang
, 
M.
Wu
, 
Y.
Chao
, 
P.
He
, 
J.
Ma
, J. Energy Chem.
2020, 43, 24;b) 
Y.
Qu
, 
M.
Shao
, 
Y.
Shao
, 
M.
Yang
, 
J.
Xu
, 
C. T.
Kwok
, 
X.
Shi
, 
Z.
Lu
, 
H.
Pan
, J. Mater. Chem. A
2017, 5, 15080.16a) 
R.
Xu
, 
J.
Huang
, 
L.
Cao
, 
L.
Feng
, 
Y.
Feng
, 
L.
Kou
, 
Q.
Liu
, 
D.
Yang
, 
L.
Feng
, J. Electrochem. Soc.
2020, 167, 026508;b) 
H.
Qi
, 
L.
Wang
, 
T.
Zuo
, 
S.
Deng
, 
Q.
Li
, 
Z.‐H.
Liu
, 
P.
Hu
, 
X.
He
, ChemElectroChem
2020, 7, 78;c) 
X.
Wen
, 
M.
Zhao
, 
Z.
Zhao
, 
X.
Ma
, 
M.
Ye
, ACS Sustainable Chem. Eng.
2020, 8, 7335.17

W.
Yang
, 
N.
Luo
, 
C.
Zheng
, 
S.
Huang
, 
M.
Wei
, Small
2019, 15, 1903904.10.1002/smll.2019039043174712518a) 
Y.
Wei
, 
D.
Lai
, 
L.
Zou
, 
X.
Ling
, 
H.
Lu
, 
Y.
Xu
, Polym. Eng. Sci.
2018, 58, 37;b) 
M. A. A.
Mohd Abdah
, 
N.
Abdul Rahman
, 
Y.
Sulaiman
, Electrochim. Acta
2018, 259, 466;c) 
N.
Khalifa
, 
A.
Souissi
, 
I.
Attar
, 
M.
Daoudi
, 
B.
Yakoubi
, 
R.
Chtourou
, Opt. Laser Technol.
2013, 54, 335;d) 
C.‐J.
Tsai
, 
Y.‐R.
Chang
, 
D.‐J.
Lee
, Ind. Eng. Chem. Res.
2018, 57, 14213.19a) 
S. K.
Saroj
, 
S.
Pal
, 
R.
Nagarajan
, Appl. Clay Sci.
2020, 185, 105411;b) 
S.
Lowum
, 
R.
Floyd
, 
R.
Bermejo
, 
J.‐P.
Maria
, J. Mater. Sci.
2019, 54, 4518;10.1063/1.509404031153285c) 
S.
Liu
, 
J.
Ji
, 
Y.
Yu
, 
H.
Huang
, Catal. Sci. Technol.
2018, 8, 4264.20

B.
Zhao
, 
W.
Ma
, 
P.
Zhang
, 
Q.
Zhang
, 
J.
Zhong
, 
H.
Matsuyama
, Chem. Pap.
2020, 74, 3913.21a) 
S.
Zhang
, 
H.
Yu
, 
Q.
Chen
, 
H.
Hu
, 
Y.
Song
, 
J.
Chen
, 
Y.
Cao
, 
M.
Xiang
, J. Polym. Res.
2020, 27, 31;b) 
S.
Li
, 
P.
Bai
, 
Y.
Li
, 
W.
Jia
, 
X.
Li
, 
Y.
Meng
, 
L.
Ma
, 
Y.
Tian
, Langmuir
2020, 36, 6765.3246049110.1021/acs.langmuir.0c0088722

E.
Kavya Valsan
, 
A.
John
, 
M.
Raghavendra
, 
H. B.
Ravikumar
, J. Electrochem. Soc.
2020, 167, 060525.23

B.
Tang
, 
L.
Shan
, 
S.
Liang
, 
J.
Zhou
, Energy Environ. Sci.
2019, 12, 3288.24

Y.
Wu
, 
Y.
Zhang
, 
Y.
Ma
, 
J. D.
Howe
, 
H.
Yang
, 
P.
Chen
, 
S.
Aluri
, 
N.
Liu
, Adv. Energy Mater.
2018, 8, 1802470.25

T.
Jiao
, 
Q.
Yang
, 
S.
Wu
, 
Z.
Wang
, 
D.
Chen
, 
D.
Shen
, 
B.
Liu
, 
J.
Cheng
, 
H.
Li
, 
L.
Ma
, 
C.
Zhi
, 
W.
Zhang
, J. Mater. Chem. A
2019, 7, 16330.26a) 
J.
Wang
, 
J.
Polleux
, 
J.
Lim
, 
B.
Dunn
, J. Phys. Chem. C
2007, 111, 14925;b) 
X.
Zhang
, 
J.
Li
, 
H.
Ao
, 
D.
Liu
, 
L.
Shi
, 
C.
Wang
, 
Y.
Zhu
, 
Y.
Qian
, Energy Storage Mater.
2020, 30, 337;c) 
D.
Cao
, 
W.
Kang
, 
W.
Wang
, 
K.
Sun
, 
Y.
Wang
, 
P.
Ma
, 
D.
Sun
, Small
2020, 16, 1907641.10.1002/smll.2019076413273469027a) 
J.
Guan
, 
Y.
Chen
, 
L.
Cao
, 
Y.
Liu
, 
P.
Lian
, 
Y.
Gao
, 
X.
Shi
, J. Power Sources
2020, 469, 228307;b) 
H.
Wang
, 
J.‐L.
Lan
, 
H.
Yuan
, 
S.
Luo
, 
Y.
Huang
, 
Y.
Yu
, 
Q.
Cai
, 
X.
Yang
, Appl. Surf. Sci.
2020, 518, 146221.28

L.
Ma
, 
S.
Chen
, 
X.
Li
, 
A.
Chen
, 
B.
Dong
, 
C.
Zhi
, Angew. Chem., Int. Ed.
2020, 59, 23836.10.1002/anie.20201178832935895

## Supporting information

Supporting Information

Supplemental Movie 1

## Data Availability

Research data are not shared.
