# Supplementary material for: A Self‐Healing Flexible Quasi‐Solid Zinc‐Ion Battery Using All‐In‐One Electrodes
Source: Adv Sci (Weinh). 2021 Feb 14;8(8):2004689. doi: 10.1002/advs.202004689 (PMC8061350; doi:10.1002/advs.202004689)
Supplement: Supplementary file 1 — Supporting Information [file ADVS-8-2004689-s002.pdf]

© 2021 Wiley-VCH GmbH

ADVANCED SCIENCE
Open Access

Supporting Information

for *Adv. Sci.,* DOI: 10.1002/advs.202004689

A Self-Healing Flexible Quasi-Solid Zinc-Ion Battery Using All-in-One Electrodes

Jinyun Liu, Jiawei Long, Zihan Shen, Xing Jin, Tianli Han, Ting Si, Huigang Zhang**

Supporting Information

A Self-Healing Flexible Quasi-Solid Zinc-Ion Battery Using All-in-One Electrodes

Jinyun Liu, Jiawei Long, Zihan Shen, Xing Jin, Tianli Han, Ting Si, Huigang Zhang**

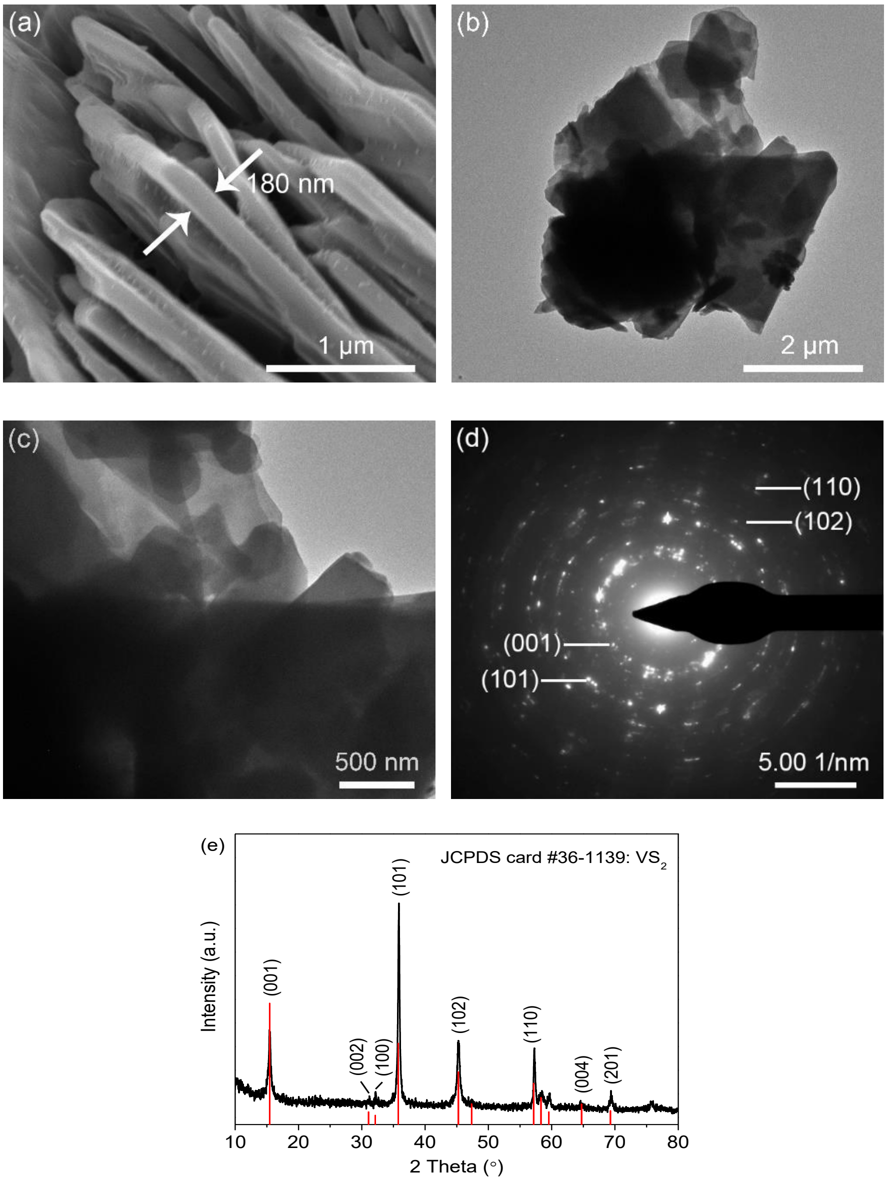

Fig. S1 (a) SEM, (b) high- and (c) low-magnification TEM images of VS_2 nanosheets. (d) The SAED pattern of the VS_2 nanosheet. (e) XRD pattern of the VS_2/CC cathode.

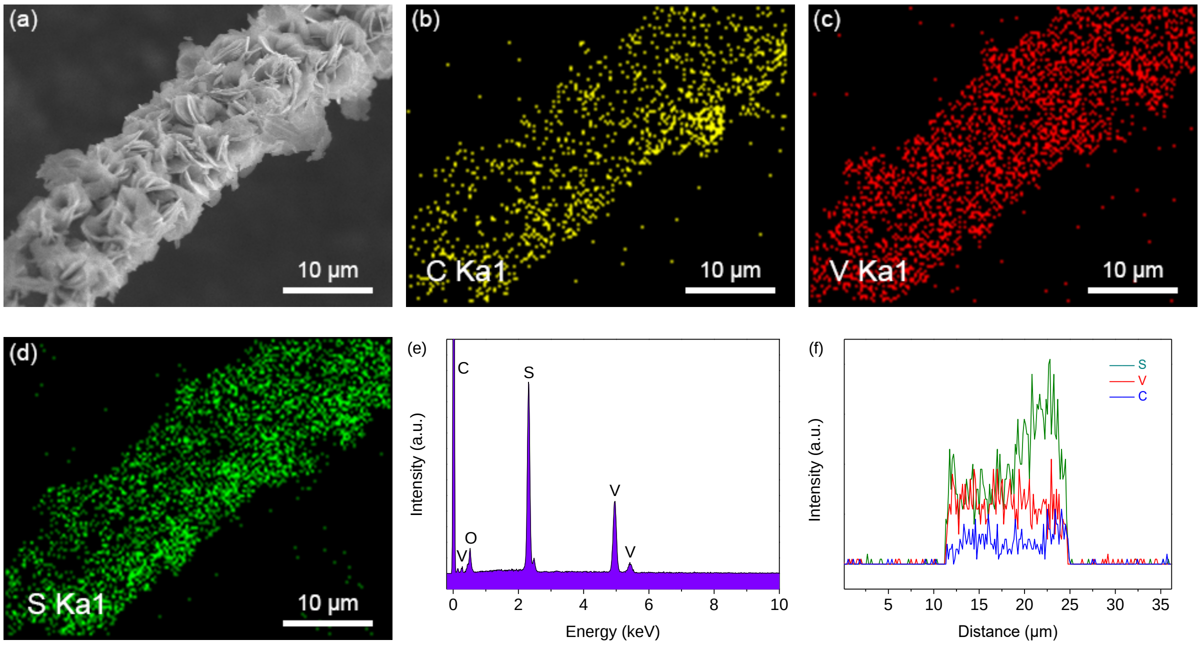

Fig. S2 (a) SEM image of VS_2/CC and corresponding mapping images of (b) C, (c) V and (d) S. (e) EDX spectrum and (f) Line-scan profiles of VS_2/CC.

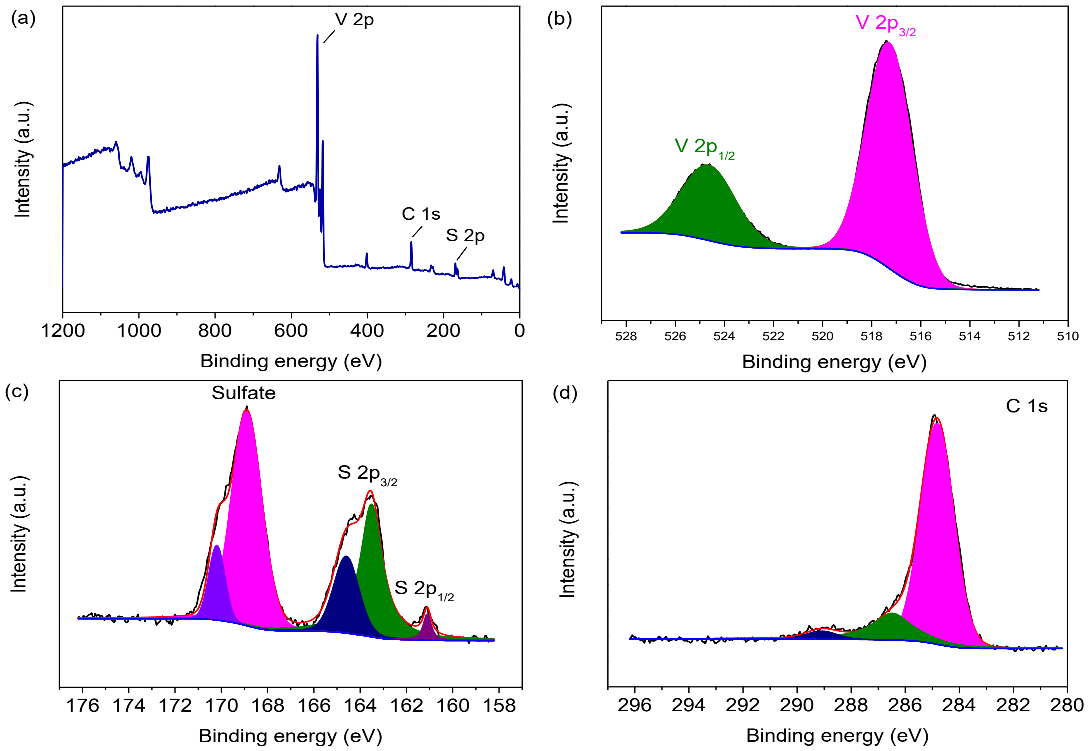

Fig. S3 (a) XPS survey spectrum of VS_2/CC. High-resolution XPS spectra of (b) V 2p, (c) S 2p, and (d) C1s.

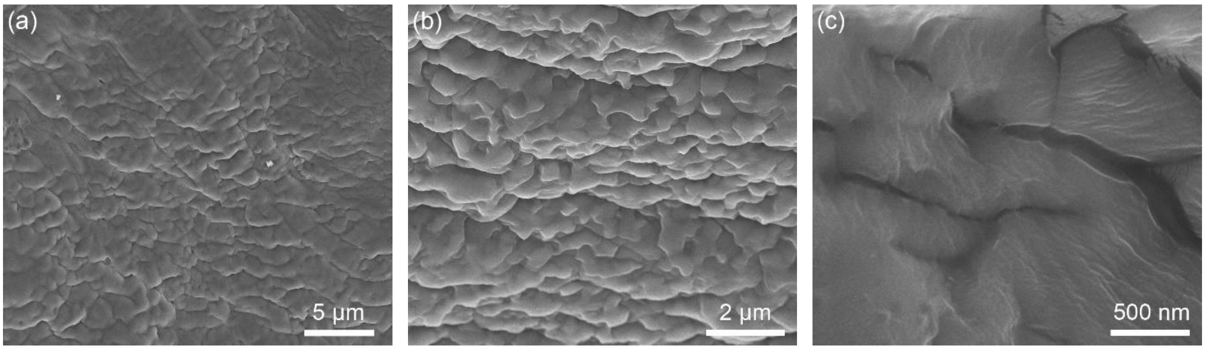

Fig. S4 SEM images of pure PVA hydrogel at different magnifications.

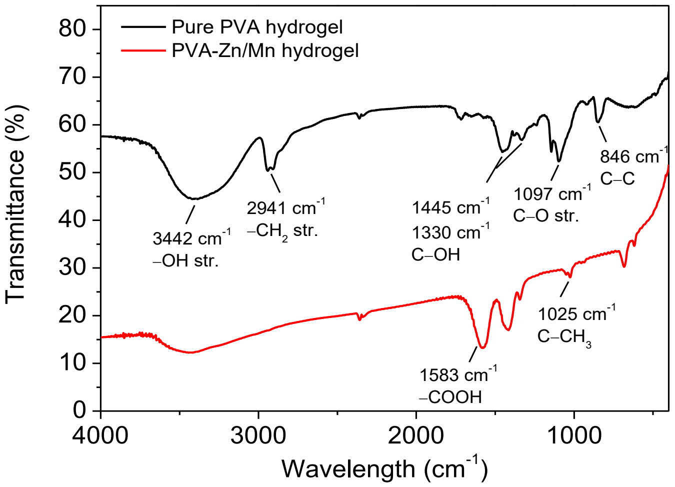

Fig. S5 FT-IR spectra of the PVA-Zn/Mn and pure PVA hydrogels.

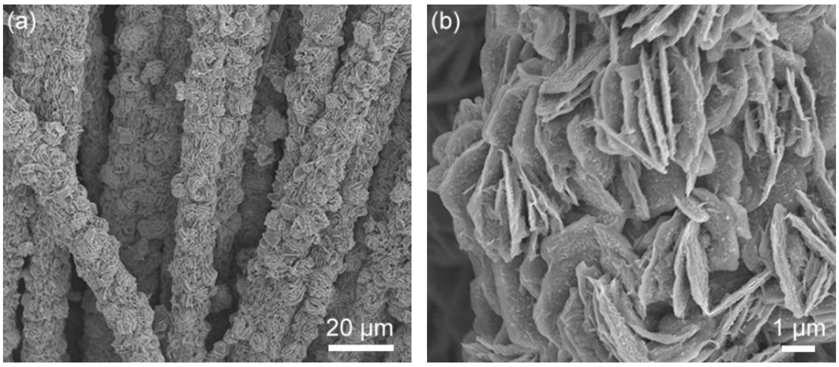

Fig. S6 (a) Low- and (b) high-magnification SEM images of the VS_2/CC cathode after cycling 40 times.

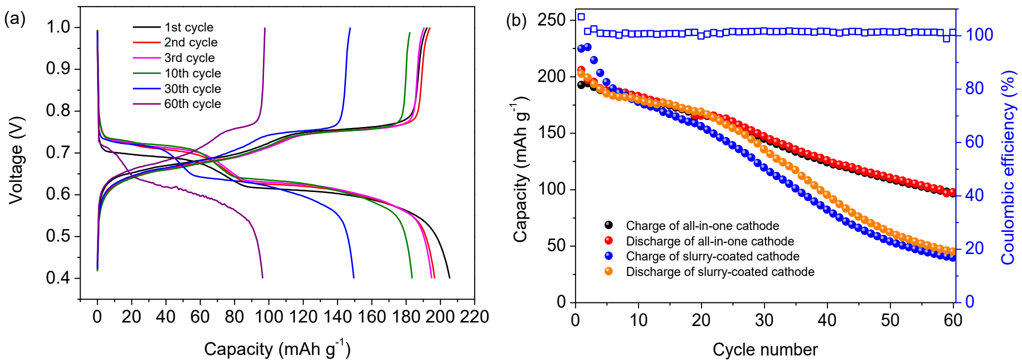

Fig. S7 (a) Charge-discharge curves of the Zn-ion battery. (b) Cycling performances of the batteries at 50 mA g^{-1} based on the all-in-one VS_2/CC and slurry-coated cathodes. The slurry-coated cathode was prepared by milling VS_2 nanosheets, carbon black, polyvinylidene fluoride (PVDF), at the mass ratio of 7:2:1, and then stirred for 4 h. The slurry was casted on carbon paper with the thickness of 100 μm and dried at 60 °C under vacuum. The electrode was cut to discs with the diameter of 12 mm. At last, the battery was assembled by following the procedure the same as the batteries using all-in-one electrodes.

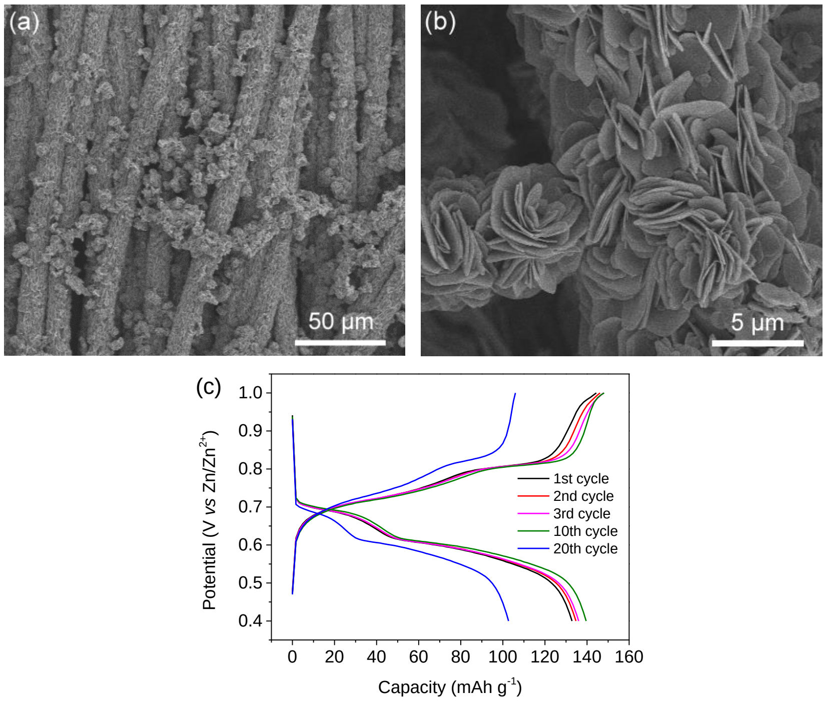

Fig. S8 (a) Low- and (b) high-magnification SEM images of the VS_2/CC cathode with a VS_2 loading of 25.3 mg cm^{-2}. The sample was prepared by using relative high concentrations of NH_4VO_3 and thioacetamide, while the other experimental procedures were the same. (c) Corresponding charge-discharge curves at 50 mA g^{-1}.

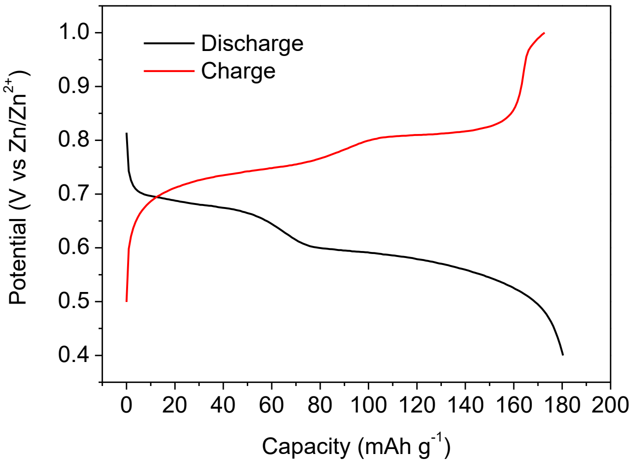

Fig. S9 Charge and discharge curves of the VS_2/CC cathode at 50 mAh g^{-1}. The Zn^{2+} intercalation number (x+y) was calculated according to the discharge curve at 50 mAh g^{-1} and based on the following equation:[1]

$$x + y = \frac{nC_aM}{C_0}$$

where n refers to the number of electron exchange, C_a=180.3 mAh g^{-1}, $M(VS_2)$=115 g mol^{-1}, C_0=26.8 nm M^{-1}, $F=N_A \times e$=96500 C mol^{-1}, 1 Ah=3600 C).

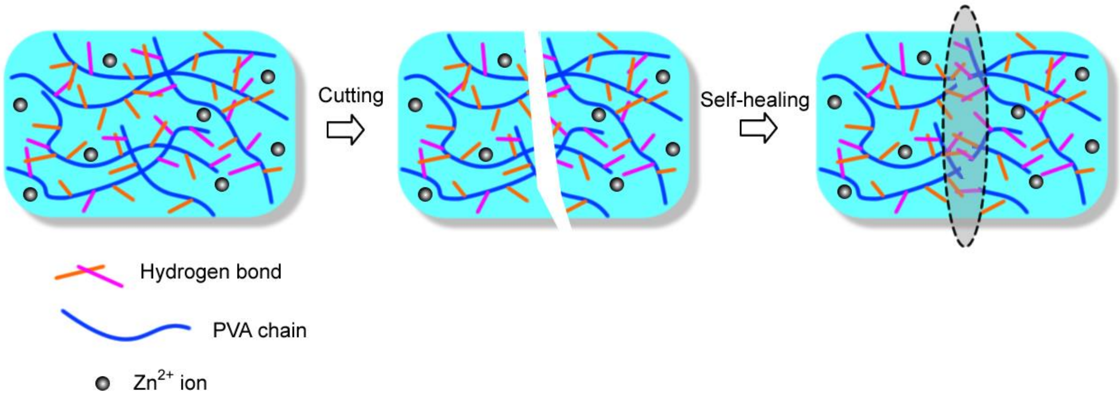

Fig. S10 Illustration for the cutting/self-healing process of the hydrogel electrolyte.

Table S1. Charge transfer resistance of VS_2/CC cathode within one charge-discharge cycle.

Battery potential (V)	Charge transfer resistance (Ω)
Fresh cell 0.9 V	23.1
Discharging to 0.8 V	24.6
Discharging to 0.7 V	43.3
Discharging to 0.6 V	171.4
Discharging to 0.5 V	170.2
Discharging to 0.4 V	117.0
Charging to 0.5 V	173.4
Charging to 0.6 V	201.7
Charging to 0.7 V	195.0
Charging to 0.8 V	51.2
Charging to 0.9 V	34.7
Charging to 1.0 V	31.2

Table S2. Comparison on the electrochemical performance of some cathode of Zn-ion batteries.

Cathode	Electrolyte	Separator	Anode	Capacity /mAh g^{-1}	Rates /A g^{-1}	Cycle numbers	Ref.
$ZnMn_2O_4$	2.0 M $ZnSO_4$ + 0.1 M $MnSO_4$	Glass fiber	Zn foil	67	0.1	40	[2]
$ZnMnCoO_4$	2.0 M $ZnSO_4$ + 0.1 M $MnSO_4$	Polymeric separator	Zn foil	39	0.2	120	[3]
$Na_3V_2(PO_4)_2F_3$	2 M $Zn(CF_3SO_3)_2$	Glass fiber	Zn foil	63.8	0.2	600	[4]
MoO_2/ Mo_2N	2 M $Zn(CF_3SO_3)_2$	Glass fiber	Zn foil	135	0.1	100	[5]
Layered MnO_2	1 M $ZnSO_4$	Glass fiber	Zn foil	97	0.1	50	[6]
Prussian blue	Ionic liquid choline acetate-water + zinc acetate	Glass fiber	Zn powder	54	0.1	50	[7]

Mn (BTC)	2.0 M $ZnSO_4$ + 0.1 M $MnSO_4$	Air-laid paper	ZIF-8@Zn	28	0.1	50	[8]
2, 3-diaminophenazine	2.0 M $ZnSO_4$	Glass fiber	Zn foil	28	0.1	200	[9]
Perylene-3,4,9,10-tetracarboxylic diimide	3.0 M $ZnSO_4$	Glass fiber	Zn foil	73	0.1	200	[10]
β-MnO_2@C	3 M $Zn(CF_3SO_3)_2$ + 0.1 M $MnSO_4$	Glass fiber	Zn foil	150	0.2	250	[11]
Mn_2O_3	3.0 M $ZnSO_4$ + 0.1 M $MnSO_4$	Glass fiber	Zn foil	30	0.2	200	[12]
MnO_2	1 M $ZnSO_4$ + 0.05 M H_2SO_4	Glass fiber	Zn foil	89.5	0.3	55	[13]
All-in-one VS_2/CC	Self-healing PVA-Zn/Mn hydrogel	-	All-in-one Zn/CC	123	0.2	40	This work

References

[1] a) P. He, M. Yan, G. Zhang, R. Sun, L. Chen, Q. An, L. Mai, *Adv. Energy Mater.* **2017**, *7*, 1601920; b) T. Jiao, Q. Yang, S. Wu, Z. Wang, D. Chen, D. Shen, B. Liu, J. Cheng, H. Li, L. Ma, C. Zhi, W. Zhang, *J. Mater. Chem. A* **2019**, *7*, 16330-16338.

[2] J. W. Lee, S. D. Seo, D. W. Kim, *J. Alloy. Compd.* **2019**, *800*, 478-482.

[3] A. Baby, B. Senthilkumar, P. Barpanda, *ACS Appl. Energy Mater.* **2019**, *2*, 3211-3219.

[4] W. Li, K. Wang, S. Cheng, K. Jiang, *Energy Storage Mater.* **2018**, *15*, 14-21.

[5] W. Xu, K. Zhao, Y. Wang, *Energy Storage Mater.* **2018**, *15*, 374-379.

[6] M. H. Alfaruqi, S. Islam, D. Y. Putro, V. Mathew, S. Kim, J. Jo, S. Kim, Y.-K. Sun, K. Kim, J. Kim, *Electrochim. Acta* **2018**, *276*, 1-11.

[7] Z. Liu, P. Bertram, F. Endres, *J. Solid State Electr.* **2017**, *21*, 2021-2027.

[8] X. Pu, B. Jiang, X. Wang, W. Liu, L. Dong, F. Kang, C. Xu, *Nano-Micro Lett.* **2020**, *12*, 152.

[9] Q. Wang, Y. Liu, P. Chen, *J. Power Sources* **2020**, *468*, 228401.

[10] N. Liu, X. Wu, Y. Zhang, Y. Yin, C. Sun, Y. Mao, L. Fan, N. Zhang, *Adv. Sci.* **2020**, *7*, 2000146.

[11] W. Jiang, X. Xu, Y. Liu, L. Tan, F. Zhou, Z. Xu, R. Hu, *J. Alloy. Compd.* **2020**, *827*, 154273.

[12] N. Liu, X. Wu, Y. Yin, A. Chen, C. Zhao, Z. Guo, L. Fan, N. Zhang, *ACS Appl. Mater. Inter.* **2020**, *12*, 28199-28205.

[13] C. Wu, H. Tan, W. Huang, W. Li, K. N. Dinh, C. Yan, W. Wei, L. Chen, Q. Yan, *Adv. Funct. Mater.* **2020**, *30*, 2003187.
